# Evolution of Physicochemical Properties and Phenolic Maturity of Vilana, Vidiano, Kotsifali and Mandilari Wine Grape Cultivars (*Vitis vinifera* L.) during Ripening

**DOI:** 10.3390/plants11243547

**Published:** 2022-12-15

**Authors:** Emmanouil Kontaxakis, Achilleas Atzemopoulos, Eleftherios Alissandrakis, Filippos Ververidis, Emmanouil Trantas

**Affiliations:** Department of Agriculture, School of Agricultural Sciences, Hellenic Mediterranean University, P.O. Box 1939, GR 71410 Heraklion, Greece

**Keywords:** phenolics, antioxidant activity, flavonols, flavanols, anthocyanins, *trans*-resveratrol, skin, seeds

## Abstract

Determining the optimum harvest time is a significant factor affecting the quality of the grapes and the wine. Monitoring the evolution of grapes’ physicochemical properties and phenolic maturity during ripening could be a valuable tool for determining the optimum harvest time. In this study, the total phenolic content, antioxidant activity, flavonols, flavanols, anthocyanins and resveratrol content were determined during the last weeks of ripening for the white cultivars Vilana and Vidiano, as well as for the red cultivars Kotsifali and Mandilari (*Vitis vinifera* L.). According to the results, an early harvest for the white cultivars and a late harvest for the red cultivars may increase the total phenolics and trans-resveratrol content in grapes and wine. An early harvest would be desirable to maintain high flavanols content and high levels of antioxidant activity in the grapes’ skin and seeds. Conversely, a late harvest for the red cultivars may be desirable to increase the total flavonols and anthocyanin content in grapes and wines.

## 1. Introduction

Grapevine cultivation and winemaking have a long history in Greece, starting from antiquity. Greece’s climate and most of its terroirs have always been among the most suitable for viticulture, hosting several remarkable varieties. Today, the cultivation of grapevines in Greece occupies over one million square meters [1], with a significant number of local (over 200) and international grape cultivars [2]. Grape production is of great economic international importance. Approximately 49.5% of world grape production is used for winemaking, 37% is consumed as fresh fruit, 8.5% as dried fruit and 5% is used to produce musts and juices [3].

Grapes contain sugars, organic acids, amino acids, proteins, phenolic and other compounds, depending on various factors, with the most important being the grapevine cultivar [4]. Phenolics are secondary metabolites related to the plant’s abiotic and biotic stress [5]. They have plenty of plant functions, such as antimicrobial properties (phytoalexins), protection from UV, and become stimulators of rhizobium symbiosis and pollen germination [6]. Plant phenolics are also widely known for their favourable properties and antioxidant activity in the human body, since they are known to function as antimicrobials, hypoglycaemics or antioxidants [6].

There has been great interest in researching phenolics in grapes, as well as in wine. Grapes are among the fruits with the highest content of phenolic compounds [7]. The phenolic profile of grapes depends on many factors, such as variety, maturity, genetic variability, microclimate, environmental stress and agricultural practices applied in the field [8,9]. About two-thirds of grape phenolics are located in the seeds, about one-third in the skin, and only a small percentage is found in the pulp [10,11]. However, phenolic concentration varies depending on the stage of ripening [12,13,14]. Polyphenol oxidase (PPO) activity is found to be correlated with the decrease in phenols [15]. This enzymatic activity has stimulated interest as it is related to the oxidation of phenols and quality alteration, mainly at the veraison stage when gene upregulation occurs [15,16].

Phenolics are separated into three groups: flavonoids [flavonols, flavanols (Flavan-3-ols), anthocyanins, tannins], non-flavonoids (benzoic and cinnamic acids) and stilbenes [6,15]. Anthocyanins are among the most important phenolic compounds located in the skin. They are extracted during winemaking and alcoholic fermentation into the wine. The most common anthocyanin in grapes is malvidin-3-glucoside, the concentration of which depends on the variety and abiotic factors during ripening [17]. Resveratrol is a natural stilbene in grapes produced by the grapevine, found almost exclusively in the skin of grapes, in response to several stress factors, such as injury, UV radiation and infections [18,19]. It has an exceptionally positive reputation for its health-related properties, such as preventing cardiovascular diseases, oxidative stress in neurodegenerative diseases and anticancer potency [20].

Phenolic maturity, which refers to the quantitative and qualitative evolution of the phenolic components, is crucial for the quality of the grapes, the wine, and the health benefits to the consumer [21,22]. Determining phenolic compounds during ripening may be the best way by which to decide the correct harvesting time and obtain the best results [23,24].

Vilana and Vidiano (white cultivars) and Kotsifali and Mandilari (red cultivars) are among the most valuable wine grape cultivars (*Vitis vinifera* L.) of Crete (Greece). They are used to produce remarkable wines, provided they are properly cultivated and harvested at the right time.

In this study, the total phenolic content, antioxidant activity, total flavanols and flavonols, anthocyanins and resveratrol content were determined at different ripening stages of the four grape cultivars (Vilana, Vidiano, Kotsifali and Mandilari) to provide an insight into the evolution of their physicochemical properties and their phenolic maturity, considered to be a valuable tool to determine the optimum harvest time.

## 2. Results and Discussion

### 2.1. Physical Properties of Grapes and must Characteristics

Berry weight and volume increased during the last weeks of ripening for Vidiano and Mandilari, but not for Vilana and Kotsifali (Table 1). This is because the growth rate of grapes decreases during the last weeks of ripening, while, depending on the cultivar, berry volume may remain constant or even decrease due to water evaporation [25]. Skin weight significantly increased in all cultivars, while the seed weight did not change for any cultivar; it is known that berry seeds acquire their final size and weight before veraison [25].

It has been established that there is a correlation between CIE-Lab color parameters and total anthocyanin content in grape skin [26]. Indeed, in this study, Pearson product-moment correlation coefficient calculation between total anthocyanins (Oenin mg/g FW) and color parameters (L*, C*, h) revealed a significantly strong negative correlation for Lightness (*r* = −0.993, *p* < 0.001) and Chroma (*r* = −0.972, *p* < 0.001), and a significantly strong positive correlation for Hue Angle (*r* = 0.991, *p* < 0.001).

Soluble solids (°Bx) significantly increased towards ripening for all cultivars due to sugar accumulation. However, total acidity remained constant in all cultivars, apart from the Mandilari, in which total acidity significantly decreased during the last weeks before harvest (Table 1).

**Figure 1:**
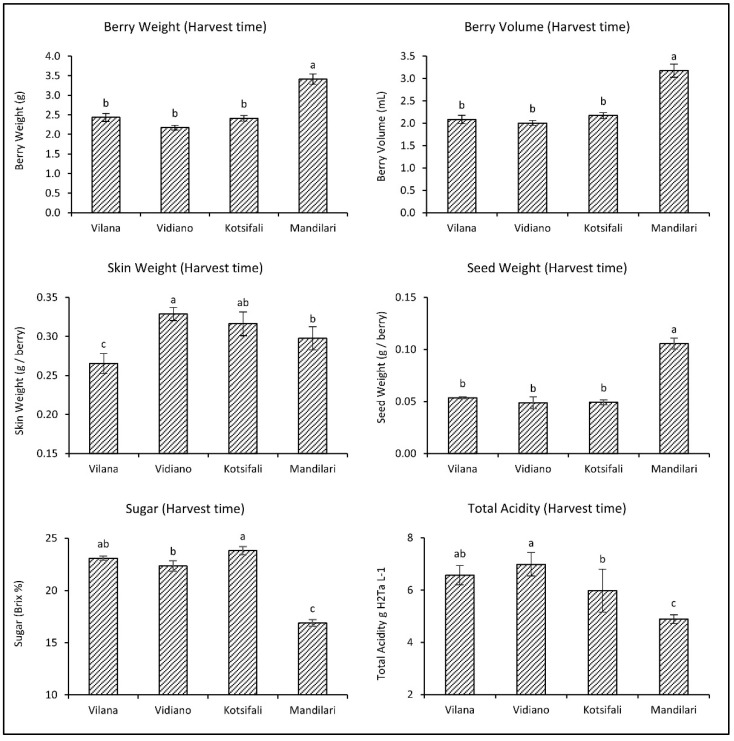
Physicochemical characteristics of Vilana, Vidiano, Kotsifali and Mandilari cultivars on harvest day. The error bars represent the standard errors of the means. Different letters indicate statistically significant differences among cultivars, according to Duncan’s multiple range test (*p* ≤ 0.05).

As it emerged from the comparative evaluation among the cultivars at harvest time, Mandilari had significantly larger berries and seeds than the other cultivars (Figure 1). Vidiano had heavier skin than Mandilari and Vilana, which had lighter skin than all cultivars. White cultivars (Vilana and Vidiano) had similar sugars (about 23 °Bx) and total acidity at harvest time. Regarding the red cultivars, Kotsifali showed significantly higher sugars than most cultivars. However, Mandilari had significantly lower sugars (only 16.9 °Bx) and total acidity than other cultivars. This inability to mature is well known for Mandilari [27] due to the grapevines’ high yield, especially when the vineyard is at a high altitude. This is the main reason why the Mandilari is traditionally co-vinificated with Kotsifali (20% and 80%, respectively), providing the PDO (Protected Designation of Origin) “Peza” and “Archanes” wines in Crete.

### 2.2. Phenolic and Antioxidants in Skin and Seeds at Harvest Time

At harvest time, the red cultivars (Kotsifali and Mandilari) had significantly more total phenolics than the white cultivars (Vilana and Vidiano) in both skin and seeds, with no differences between the red or the white cultivars (Table 2 and Table 3). Similar total phenolic content has been recently found in Kotsifali and Mandilari skin and seeds by Biniari et al. [28]. Antioxidant activity of the skin was similar among all the cultivars at harvest time (Table 2). However, regarding the seeds, Vidiano had higher antioxidant activity than Vilana and Kotsifali, which had significantly less antioxidant activity than most other cultivars. Between red cultivars, Mandilari seeds had significantly higher antioxidant activity (Table 3). As expected, the skin of the red cultivars had significantly more anthocyanins than the white cultivars [29]. Vidiano had higher skin content of flavanols than Vilana and higher seed content than all the other cultivars. Kotsifali skin had higher flavonols content than Vilana and Vidiano, while Vilana seeds had the highest flavonols content and Vidiano seeds had the least (Table 2 and Table 3). Mandilari skin had higher trans-resveratrol content than all the other cultivars, followed by Kotsifali. Several factors affect resveratrol content, such as the cultivar, abiotic and biotic factors, particularly stress factors (injuries, UV irradiation, infections, etc.). However, it has not been associated with the color of the cultivar, although red wines have a higher amount of resveratrol due to the maceration taking place in the vinification process [30,31,32]. Still, in the present study, the white cultivars had significantly lower trans-resveratrol content in their skin than the red cultivars (Table 2).

### 2.3. Evolution of Total Phenolic in Skin and Seeds during Ripening

**Figure 2:**
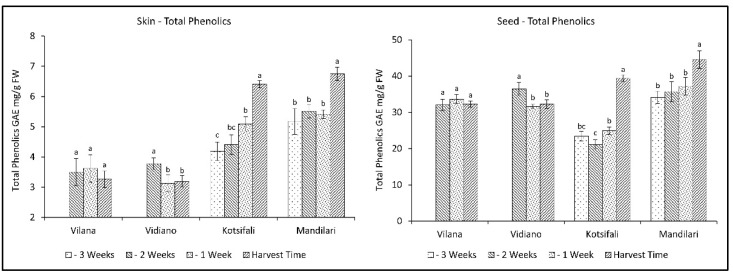
Skin and seeds total phenolics of Vilana, Vidiano, Kotsifali and Mandilari cultivars during ripening. The error bars represent the standard errors of the means. Different letters indicate statistically significant differences among weeks, according to Duncan’s multiple range test (*p* ≤ 0.05).

The evolution of total phenolics followed corresponding patterns between skin and seeds in all cultivars. The skin of white cultivars had smaller amounts of total phenolics than the red cultivars, while the seeds of all cultivars had significantly higher concentrations of total phenolics than the skins (Figure 2). This is in accordance with similar studies referring to higher phenolic content in red than in white grapes, and in the seeds than in the skins [33,34]. The red cultivars, Kotsifali and Mandilari, showed an increase in the total phenolics during ripening, reaching 6.41 and 6.75 mg g^−1^ FW of skin (GAE), as well as 39.37 and 45.53 mg g^−1^ FW of seeds (GAE), respectively. No increase in total phenolics was observed in the white varieties, but, instead, a decrease was observed in the skin and seeds of Vidiano. The increased rate of red cultivars could be attributed to the biosynthesis of anthocyanins during ripening [29,34]. According to the results, an early harvest for the white cultivars and a late harvest for the red cultivars may result in an increase in the content of total phenolics in wine.

### 2.4. Evolution of Antioxidant Activity in Skin and Seeds during Ripening

**Figure 3:**
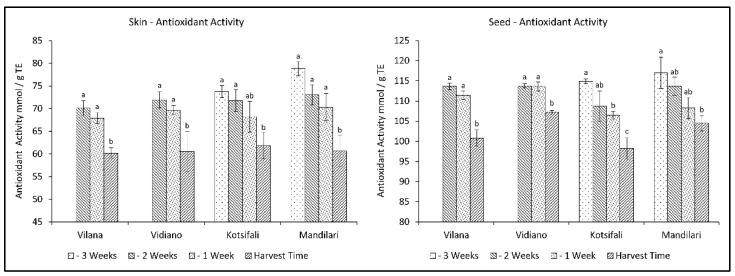
Skin and seed antioxidant activity of Vilana, Vidiano, Kotsifali and Mandilari cultivars during ripening. The error bars represent the standard errors of the means. Different letters indicate statistically significant differences among weeks, according to Duncan’s multiple range test (*p* ≤ 0.05).

Antioxidant activity has been associated in the past with the content of phenolic substances. However it depends, to a large extent, on quantitative differences in phenolic compounds [35], as well as the content of various other antioxidant substances, such as Vitamin C [36] and carotenoids [37], which decrease during ripening. In this study, antioxidant activity (AA) significantly decreased during the last weeks before harvest in a similar manner for all cultivars, in both skin and seeds. The decrease in antioxidant activity in the skin was initially limited, while it rapidly increased at ripening, reaching the same level of antioxidant activity for all cultivars at harvest time (Table 2). The same pattern was observed in seeds for white varieties. However, the red cultivars presented a gradual reduction towards ripening (Figure 3). This is in accordance with previous studies [38,39], referring to a reduction of antioxidant activity of grapes during different stages of maturation until harvest. Therefore, this study shows that an early harvest for all cultivars studied would be desirable to maintain high levels of antioxidants.

### 2.5. Evolution of Anthocyanins in Skin during Ripening

**Figure 4:**
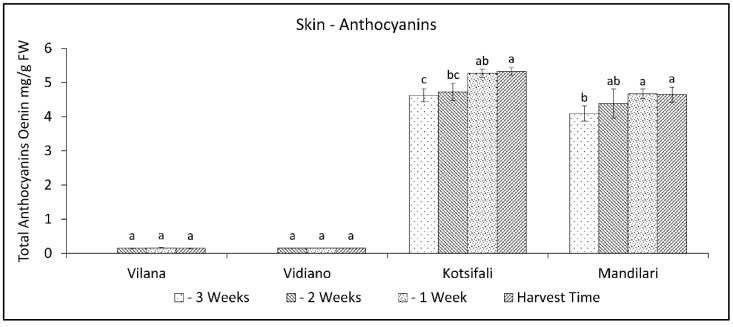
Skin anthocyanins content of Vilana, Vidiano, Kotsifali and Mandilari cultivars during ripening. The error bars represent the standard errors of the means. Different letters indicate statistically significant differences among weeks, according to Duncan’s multiple range test (*p* ≤ 0.05).

It has been established that anthocyanin content in the skin significantly depends on the cultivar [40], but it is also affected by abiotic factors, and the results are different from time to time [41]. That fact creates a significant variation in anthocyanin concentration between studies. In our study, the total anthocyanin content of Kotsifali and Mandilari significantly increased towards the last weeks of ripening (Figure 4). A similar increase in the anthocyanin content of Kotsifali and Mandilari cultivars during ripening was also found by Lanaridis and Bena-Tzourou [42]. However, although Mandilari usually has a higher content of anthocyanins compared to Kotsifali [42,43], in our study, similar anthocyanin content was found between these two red cultivars at harvest time (Table 2). The limited increase of anthocyanins in Mandilari could be attributed to the low sugar content at harvest time. It is known that sugars enhance anthocyanin production in grape skin cells and the expression of enzymes of the phenylpropanoid pathway [41,44]. As expected, anthocyanin content in the skin of white cultivars was at low levels, while no change was observed during the ripening period.

### 2.6. Evolution of Total Flavanols (Flavan-3-ols) in Skin and Seeds during Ripening

**Figure 5:**
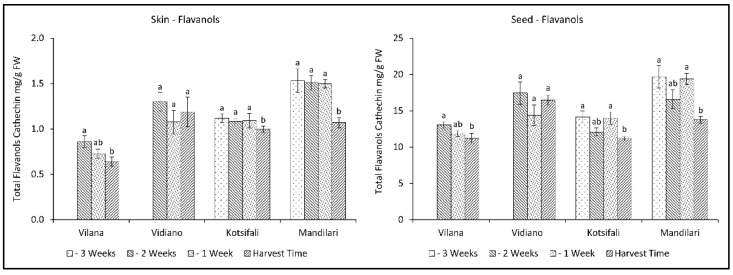
Skin and seed total flavanols content (mg CE g^−1^ FW) of Vilana, Vidiano, Kotsifali and Mandilari cultivars during ripening. The error bars represent the standard errors of the means. Different letters indicate statistically significant differences among weeks, according to Duncan’s multiple range test (*p* ≤ 0.05).

Total flavanols significantly decreased during the last weeks before harvest, in both skin and seeds, in all cultivars except Vidiano, which had the most flavanols at harvest time (Figure 5, Table 2 and Table 3). Mandilari maintained a high amount of total flavanols until the last week before harvest, when a significant reduction was observed. A similar reduction pattern was observed in all cultivars for both skin and seeds. However, a significantly higher flavanol content was found in the seeds than in the skin. It is known that the primary source of flavanols in grapes is the seeds, as well as the skin, but to a lesser extent [45,46]. Therefore, according to the results, an early harvest shortly before maturation would be desirable to maintain high flavanol content in grape skin and seeds.

### 2.7. Evolution of Total Flavonols in Skin and Seeds during Ripening

**Figure 6:**
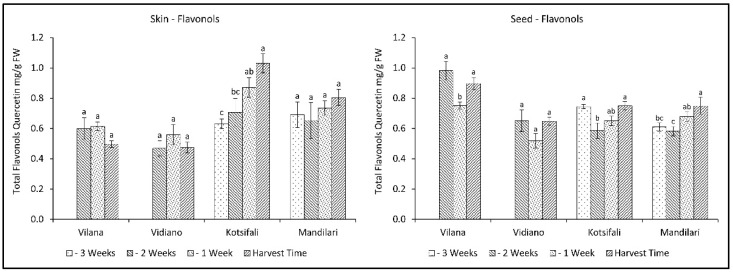
Skin and seed total flavonols content (mg QE g^−1^ FW) of Vilana, Vidiano, Kotsifali and Mandilari cultivars during ripening. The error bars represent the standard errors of the means. Different letters indicate statistically significant differences among weeks, according to Duncan’s multiple range test (*p* ≤ 0.05).

Regarding the total flavonols of skin, only Kotsifali showed a significant increase towards ripening, reaching 1.03 mg QE g^−1^ skin (FW), while no variation was observed in the other cultivars (Figure 6). Interestingly, seed flavonols decreased in the second week of sampling, and then an upward trend was observed. Despite the statistically significant changes during the last weeks of ripening, total flavonols of seeds remained virtually unchanged. However, there appears to be a tendency for flavonols in skin and seeds to increase during ripening, especially in the red cultivars. This is in agreement with previous studies, reporting an increase in flavonols in other red cultivars during grape ripening [47,48].

### 2.8. Evolution of Trans-Resveratrol in the Skin during Ripening

**Figure 7:**
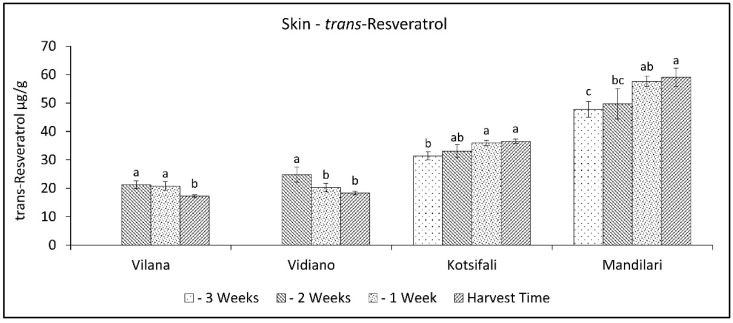
Skin trans-resveratrol content (mg g^−1^ skin FW) of Vilana, Vidiano, Kotsifali and Mandilari cultivars during ripening. The error bars represent the standard errors of the means. Different letters indicate statistically significant differences among weeks, according to Duncan’s multiple range test (*p* ≤ 0.05).

A different pattern of trans-resveratrol variation was observed between the white and the red cultivars. During the last weeks of ripening, trans-resveratrol decreased in white cultivars, while in red cultivars, it increased (Figure 7). Vilana and Vidiano had significantly lower trans-resveratrol content at harvest time than the red cultivars, while between red cultivars, Mandilari had significantly higher trans-resveratrol content (Table 2). Resveratrol accumulation in grapes is known to be directly linked to several stress factors [30]. However, in this study, where all cultivars were grown in the same conditions, there seemed to be a correlation with the degree of ripeness, but also depending on the cultivar [49]. Moreover, as mentioned above, the white cultivars had significantly lower trans-resveratrol content in their skin than the red cultivars. Therefore, as the results show, resveratrol concentration in the white cultivars studied benefits from an early harvest and, in the red cultivars, from a late harvest.

## 3. Materials and Methods

### 3.1. Sample Preparation

The study was carried out in a 25-year-old vineyard in Heraklion Prefecture, Crete (Greece), at around 500 m altitude. The grapevines of the four cultivars studied (Vilana, Vidiano, Kotsifali and Mandilari) were all grafted onto 1103P rootstock and planted, in the same vineyard, at 1.30 m on the lines and 2.40 m between the lines.

Sampling was simultaneously started for all four cultivars and were performed once a week. Three samplings were conducted for white cultivars and four for the red cultivars until harvest. The harvest time was determined, according to the usual practice, based on the potential alcoholic strength of wines. Skins and seeds were carefully removed from grapes, washed with distilled water and dried on absorbent paper. One gram of fresh skin or seeds was smashed and extracted with 10 mL of acidified methanol (MeOH:HCl 99:1), by shaking on a mechanical shaker at 4 °C in the dark for 24 h. After extraction, the extracts were filtered with syringe filters of 0.45 μm (Puradisk, Whatman, Maidstone, United Kingdom) and kept at −18 °C until analysis. Four sample replicates were analyzed for each of the four cultivars for each sampling. All analyses were carried out in triplicate.

### 3.2. Physical Properties of Grapes and must Characteristics

For each sampling and each cultivar, the weight and the volume of the berries were measured, as well as the weight of the skin and the seeds (average of 100 berries). Color parameters [Lightness (L*), Chroma (C*) and Hue Angle (h)] of the berries were evaluated using the CIE-Lab color system [50], with a Minolta Chroma Meter (Konica Minolta CR-400, Tokyo, Japan). Sugars (°Bx) of the grape must were measured using a digital refractometer (Atago, Tokyo, Japan). Titratable acidity (g H_2_Ta L^−1^) was determined by titration with NaOH (0,1 M), and pH was measured using a HI2020 pH meter (Hanna, Woonsocket, RI, USA).

### 3.3. Total Phenolic Content

Total phenolic content was determined using the Folin-Ciocalteu’s method [51]. A sample of 50 μL was combined with 2 mL of Na_2_CO_3_ (2% *w/v*). After 2 min, 0.1 mL Folin-Ciocalteu reagent was added, and the mixture was left for 30 min in the dark. The absorbance of the mixture was determined at 765 nm using a Shimadzu UV-1800 Spectrophotometer (Shimadzu, Tokyo, Japan) with water as a blank. The concentration of total phenolic content was estimated from a calibration curve constructed by plotting known solutions of gallic acid (0.0625–1 mg mL^−1^) against A765 (R^2^ = 0.9999). Results were expressed as mg g^−1^ gallic acid equivalents (GAE) on a fresh weight basis (FW).

### 3.4. Total Anthocyanin Content

The color of red grapes results from the accumulation of anthocyanins, which are usually only localized in the skin of the berry [29]. The total anthocyanin content was determined according to a modified method by Poudel et al. [52]. An aliquot of skin extracts, suitably diluted as appropriate to have an absorbance between 0.3 and 0.7, was read at 537 nm using a Shimadzu UV-1800 Spectrophotometer (Shimadzu, Tokyo, Japan). The total anthocyanin content was estimated from a calibration curve constructed by plotting known solutions of Oenin chloride (Extrasynthese, Genay, France) (0.03125–0.5 mg mL^−1^) against A537 (R^2^ = 1). Results were expressed as mg g^−1^ oenin equivalents (OE) on a fresh weight basis (FW).

### 3.5. Total Flavanols (Flavan-3-ols)

The total flavanol content was estimated, according to Arnous et al. [53], using the p-dimethylaminocinnamaldehyde (DMACA) method. A portion of 0.2 mL of skin or seed extract, diluted 1:100 with MeOH, was placed in a test tube and 1 mL of DMACA solution (0.1% in acidified methanol) was added. The mixture was vortexed and allowed to react at room temperature for 10 min. The absorbance at 640 nm was measured against a blank solution without DMACA. The concentration of total flavanol content was estimated from a calibration curve constructed by plotting known dilutions of catechin (0.5–16 mg L^−1^) against A640 (R^2^ = 1). Results were expressed as mg g^−1^ catechin equivalents (CE) on a fresh weight basis (FW).

### 3.6. Total Flavonols

The total flavonol content was estimated according to a modified method by Poudel, Tamura, Kataoka and Mochioka [52]. A portion of 0.25 mL of skin or seed extracts diluted (1:10) with 10% ethanol was placed in a test tube and 0.25 mL 0.1% HCl in 95% ethanol (*v*/*v*) and 4.55 mL 2% HCl (*v*/*v*) were added. The mixture was vortexed and allowed to react at room temperature for 15 min before reading the absorbance at 360 nm using a Shimadzu UV-1800 Spectrophotometer (Shimadzu, Tokyo, Japan). The concentration of total flavonol content was estimated from a calibration curve constructed by plotting known solutions of quercetin (0.0625–1 mg mL^−1^) against A360 (R^2^ = 0.9998). Results were expressed as mg g^−1^ quercetin equivalents (QE) on a fresh weight basis (FW).

### 3.7. Antioxidant Activity

The total antioxidant activity was determined according to the DPPH (2,2-diphenyl-L-picrylhydrazyl) radical scavenging spectrophotometric method [52]. An aliquot of 25 μL of diluted (1:10) sample was mixed with 975 μL DPPH solution (60 μM in MeOH). The mixture was vortexed, and the absorbance was read at 515 nm, using methanol as a blank, at t = 0 and after 30 min stored in the dark. The total antioxidant activity was estimated from a calibration curve constructed by plotting known solutions of Trolox against A515 (R^2^ = 0.9999). Results were expressed as mmol g^−1^ Trolox equivalent antioxidant capacity (TEAC) on a fresh weight basis (FW).

### 3.8. Resveratrol Content

Resveratrol analysis was performed using the Reversed-phase High-Performance Liquid Chromatography method [54]. The HPLC system consisted of an Agilent 1200 Degasser (G1379B), an Agilent 1200 Bin Pump (G1312A), an Agilent 1200 Autosampler (G1329A), an Agilent 1200 Thermostatted Column Compartment (G1316A) and an Agilent 1200 Diode Array Detector (G1315D) (Agilent, Santa Clara, CA, USA). Before analysis, skin and seed extracts were filtered with syringe filters of 0.45 μm (Puradisk, Whatman, Maidstone, United Kingdom). The samples were separated using an Agilent Zorbax Eclipse XDB-C18 column (150 mm × 4.6 mm, 5 μm). The mobile phase consisted of methanol (HPLC grade), the column temperature was set at 35 °C, the injection volume was 20 μL, and the flow rate was 1 mL min^−1^. The detection of resveratrol was performed at 306 nm. The run time for samples was 10 min, with resveratrol being detected at about 2.3 min. Several standard solutions from 1.25 to 40 mg L^−1^ were used to construct a calibration curve (R^2^ = 1). Results were expressed as mg g^−1^ on a fresh weight basis (FW).

### 3.9. Statistical Analysis

One-way ANOVA, Duncan’s Multiple Range Test, and Pearson product-moment correlation coefficient calculation were performed using the Statistical Programme for Society Sciences (IBM SPSS Statistics, version 19).

## 4. Conclusions

Determining the optimum harvest time is a significant factor affecting the quality of the grapes and the wine. In addition to defining the technological ripening of the grapes, determining phenolic compounds during ripening may be the best way to decide the correct harvesting time and obtain the best results. Phenolic maturity is crucial for the quality of the grapes, the wine, and the health benefits to the consumer [20]. As emerged from the present study, total phenolic content, antioxidant activity, flavonols, flavanols, anthocyanins and resveratrol content varies during ripening, especially near harvest.

In the present study, the evolution of total phenolics followed corresponding patterns between skin and seeds in all cultivars. The red cultivars, Kotsifali and Mandilari, increased the total phenolics during ripening. No increase in total phenolics was observed in the white varieties, but, instead, a decrease was observed in the skin and seeds of Vidiano. According to the results, an early harvest for the white cultivars and a late harvest for the red cultivars may increase the content of total phenolics in grapes and wine.

Antioxidant activity significantly decreased during the last weeks before harvest, for all cultivars, in both skin and seeds, especially in the last week before harvest. Therefore, it appears that an early harvest, for all of the cultivars studied, is desirable to maintain high levels of antioxidants, provided, of course, that technological maturity has also been achieved.

Regarding anthocyanin content, as expected, the skin of the red cultivars had significantly more anthocyanins, with an upward trend, than the white cultivars, which had even less than the seeds. A late harvest of the red cultivars may be desirable to increase anthocyanin content in wines. However, it is not a factor that should be considered in white cultivars.

Total flavanols significantly decreased during the last weeks before harvest, in both skin and seeds, in all cultivars except Vidiano. Indeed, a significant reduction was observed in the last week before harvest. Therefore, an early harvest shortly before maturation would be desirable to maintain high flavanols content in the grapes’ skin and seeds.

Regarding total flavonols in grape skin and seeds, there seems to be a tendency to increase during the last weeks of ripening, especially in the red varieties. Thus, a late harvest could potentially benefit the total flavonols content for the red cultivars studied.

Finally, during the last weeks of ripening, trans-resveratrol content decreased in white cultivars, while in red cultivars it increased. Indeed, white cultivars had significantly lower trans-resveratrol content at harvest time than red cultivars. Resveratrol content in the grapes’ skin seems to be correlated with the degree of ripeness, with white varieties benefiting from an early harvest and red varieties from a late harvest.

The results of this study demonstrate the importance of monitoring the evolution of grape physicochemical properties and phenolic maturity during ripening to determine the optimum harvest time.

## Figures and Tables

**Table 1:** Physical properties, berry color and must characteristics of Vilana, Vidiano, Kotsifali and Mandilari cultivars during the last weeks before harvest.

			Physical Properties of Berries							Must Characteristics		
Cultivar		Period before Harvest (week)	Berry Weight (g)	Berry Volume (mL)	Seed Weight (g/berry)	Skin Weight (g/berry)	Lightness (L*)	Chroma (C*)	Hue Angle (h)	Soluble Solids (Brix %)	Total Acidity (g H_2_Ta/L)	pH
White cultivars	Vilana	2	2.23 ^a^	2.09 ^a^	0.052 ^a^	0.226 ^b^	42.20 ^a^	14.18 ^a^	100.4 ^a^	20.20 ^c^	7.13 ^a^	3.46 ^b^
		1	2.53 ^a^	2.05 ^a^	0.052 ^a^	0.279 ^a^	43.12 ^b^	11.80 ^b^	98.0 ^b^	21.48 ^b^	6.58 ^a^	3.70 ^a^
		0	2.43 ^a^	2.09 ^a^	0.053 ^a^	0.265 ^a^	42.14 ^a^	12.04 ^b^	90.2 ^c^	23.08 ^a^	6.58 ^a^	3.71 ^a^
	Vidiano	2	1.88 ^b^	1.75 ^b^	0.047 ^a^	0.280 ^b^	41.47 ^a^	15.04 ^a^	105.2 ^b^	19.28 ^b^	7.32 ^a^	3.42 ^c^
		1	2.21 ^a^	1.89 ^ab^	0.051 ^a^	0.297 ^a^	41.73 ^a^	11.59 ^b^	109.1 ^a^	20.38 ^b^	7.03 ^a^	3.61 ^b^
		0	2.17 ^a^	2.01 ^a^	0.048 ^a^	0.328 ^a^	41.09 ^a^	10.33 ^c^	106.3 ^b^	22.35 ^a^	7.00 ^a^	3.89 ^a^
Red cultivars	Kotsifali	3	2.14 ^a^	2.00 ^a^	0.047 ^a^	0.229 ^b^	26.89 ^b^	4.01 ^a^	338.4 ^a^	18.85 ^b^	6.50 ^a^	3.58 ^b^
		2	2.43 ^a^	2.25 ^a^	0.052 ^a^	0.295 ^a^	28.34 ^a^	3.50 ^b^	343.5 ^a^	20.05 ^b^	7.01 ^a^	3.52 ^b^
		1	2.37 ^a^	2.25 ^a^	0.047 ^a^	0.278 ^a^	25.58 ^c^	2.32 ^c^	346.9 ^a^	23.28 ^a^	5.81 ^a^	3.67 ^b^
		0	2.41 ^a^	2.17 ^a^	0.049 ^a^	0.316 ^a^	26.22 ^bc^	2.37 ^c^	338.9 ^a^	23.80 ^a^	5.98 ^a^	4.07 ^a^
	Mandilari	3	3.01 ^b^	2.85 ^b^	0.114 ^a^	0.279 ^b^	27.66 ^b^	4.31 ^a^	331.4 ^a^	12.63 ^c^	8.15 ^a^	3.09 ^b^
		2	3.06 ^b^	3.20 ^a^	0.104 ^a^	0.310 ^a^	29.81 ^a^	4.19 ^a^	353.8 ^a^	14.48 ^b^	6.19 ^b^	3.57 ^a^
		1	3.05 ^b^	3.29 ^a^	0.106 ^a^	0.299 ^a^	26.43 ^c^	2.56 ^b^	340.8 ^a^	15.53 ^b^	6.09 ^b^	3.54 ^a^
		0	3.41 ^a^	3.18 ^a^	0.105 ^a^	0.297 ^a^	26.70 ^bc^	2.47 ^b^	326.3 ^a^	16.90 ^a^	4.89 ^c^	3.61 ^a^

Different letters indicate statistically significant differences among weeks, according to Duncan’s multiple range test (*p* ≤ 0.05).

**Table 2:** Phenolic content, antioxidant activity, anthocyanins, total flavanols, total flavonols and trans-resveratrol content in the skin of Vilana, Vidiano, Kotsifali and Mandilari cultivars on harvest day.

Cultivar	Total Phenolics (Gallic Acid mg/g FW)	Antioxidant Activity (Trolox mmol/g FW)	Total Anthocyanins (Oenin mg/g FW)	Total Flavanols (Catechin mg/g FW)	Total Flavonols (Quercetin mg/g FW)	*trans*-Resveratrol (μg/g FW)
Vilana	3.26 ^b^	60.17 ^a^	0.148 ^b^	0.640 ^b^	0.496 ^b^	17.24 ^c^
Vidiano	3.19 ^b^	60.55 ^a^	0.150 ^b^	1.187 ^a^	0.474 ^b^	18.31 ^c^
Kotsifali	6.41 ^a^	61.84 ^a^	5.324 ^a^	0.995 ^ab^	1.030 ^a^	36.58 ^b^
Mandilari	6.75 ^a^	60.63 ^a^	4.645 ^a^	1.069 ^ab^	0.804 ^ab^	59.08 ^a^

Different letters indicate statistically significant differences among cultivars, according to Duncan’s multiple range test (*p* ≤ 0.05).

**Table 3:** Phenolic content, antioxidant activity, total flavanols and total flavonols content in seeds of Vilana, Vidiano, Kotsifali and Mandilari cultivars on harvest day.

Cultivar	Total Phenolics (Gallic Acid mg/g FW)	Antioxidant Activity (Trolox mmol/g FW)	Total Flavanols Catechin mg/g FW)	Total Flavonols (Quercetin mg/g FW)
Vilana	32.30 ^b^	100.78 ^bc^	11.20 ^c^	0.897 ^a^
Vidiano	32.26 ^b^	107.29 ^a^	16.47 ^a^	0.648 ^c^
Kotsifali	39.37 ^a^	98.24 ^c^	11.18 ^c^	0.750 ^b^
Mandilari	44.53 ^a^	104.49 ^ab^	13.76 ^b^	0.749 ^b^

Different letters indicate statistically significant differences among cultivars, according to Duncan’s multiple range test (*p* ≤ 0.05).

## Data Availability

The data presented in this study are available on request from the corresponding author.

## References

[1] Eurostat Vineyards in the EU—Statistics. https://ec.europa.eu/eurostat/statistics-explained/index.php?title=Vineyards_in_the_EU_-_statistics.

[2] Koufos G.C., Mavromatis T., Koundouras S., Jones G.V. (2020). Adaptive capacity of winegrape varieties cultivated in Greece to climate change: Current trends and future projections. OENO One.

[3] FAO, OIV Table and Dried Grapes. http://www.fao.org/3/a-i7042e.pdf.

[4] Cosme F., Gonçalves B., Inês A., Jordão A.M., Vilela A., Morata A., Loira I. (2016). Grape and wine metabolites: Biotechnological approaches to improve wine quality. Grape and Wine Biotechnology.

[5] Shalaby S., Horwitz B.A. (2015). Plant phenolic compounds and oxidative stress: Integrated signals in fungal–plant interactions. Curr. Genet..

[6] Ververidis F., Trantas E., Douglas C., Vollmer G., Kretzschmar G., Panopoulos N. (2007). Biotechnology of flavonoids and other phenylpropanoid-derived natural products. Part I: Chemical diversity, impacts on plant biology and human health. Biotechnol. J..

[7] Xia E.-Q., Deng G.-F., Guo Y.-J., Li H.-B. (2010). Biological Activities of Polyphenols from Grapes. Int. J. Mol. Sci..

[8] Blancquaert E., Oberholster A., Ricardo-da-Silva J., Deloire A. (2019). Effects of abiotic factors on phenolic compounds in the Grape Nerry-a review. S. Afr. J. Enol. Vitic..

[9] Rienth M., Vigneron N., Darriet P., Sweetman C., Burbidge C., Bonghi C., Walker R.P., Famiani F., Castellarin S.D. (2021). Grape Berry Secondary Metabolites and Their Modulation by Abiotic Factors in a Climate Change Scenario–A Review. Front. Plant Sci..

[10] Teixeira A., Eiras-Dias J., Castellarin S.D., Gerós H. (2013). Berry phenolics of grapevine under challenging environments. Int. J. Mol. Sci..

[11] Zhu Y.-Y., Zhao P.-T., Wang X.-Y., Zhang J., Wang X.-H., Tian C.-R., Ren M.-M., Chen T.-G., Yuan H.-H. (2019). Evaluation of the potential astringency of the skins and seeds of different grape varieties based on polyphenol/protein binding. Food Sci. Technol..

[12] Pirie A.J., Mullins M.G. (1980). Concentration of phenolics in the skin of grape berries during fruit development and ripening. Am. J. Enol. Vitic..

[13] Allegro G., Pastore C., Valentini G., Filippetti I. (2021). The Evolution of Phenolic Compounds in Vitis vinifera L. Red Berries during Ripening: Analysis and Role on Wine Sensory—A Review. Agronomy.

[14] Blancquaert E.H., Oberholster A., Ricardo-da-Silva J.M., Deloire A.J. (2019). Grape flavonoid evolution and composition under altered light and temperature conditions in Cabernet Sauvignon (*Vitis vinifera* L.). Front. Plant Sci..

[15] Awad M.A., Al-Qurashi A.D., Alrashdi A.M., Mohamed S.A., Faidi F. (2017). Developmental changes in phenolic compounds, antioxidant capacity and enzymes activity in skin of ‘El-Bayadi’table grapes. Sci. Hortic..

[16] Yoruk R., Marshall M.R. (2003). Physicochemical properties and function of plant polyphenol oxidase: A review. J. Food Biochem..

[17] He F., Mu L., Yan G.-L., Liang N.-N., Pan Q.-H., Wang J., Reeves M.J., Duan C.-Q. (2010). Biosynthesis of Anthocyanins and Their Regulation in Colored Grapes. Molecules.

[18] Stervbo U., Vang O., Bonnesen C. (2007). A review of the content of the putative chemopreventive phytoalexin resveratrol in red wine. Food Chem..

[19] Mavrakis T., Agalias A., Skaltsounis A., Ververidis F. (2008). Application of bioactive plant substances from olive tissues and grapes pomace in non-chemical disease control. Planta Med..

[20] Kontaxakis E., Trantas E., Ververidis F. (2020). Resveratrol: A Fair Race Towards Replacing Sulfites in Wines. Molecules.

[21] Kontoudakis N., Esteruelas M., Fort F., Canals J.M., De Freitas V., Zamora F. (2011). Influence of the heterogeneity of grape phenolic maturity on wine composition and quality. Food Chem..

[22] Cagnasso E., Rolle L., Caudana A., Gerbi V. (2008). Relationship between grape phenolic maturity and red wine phenolic composition. Ital. J. Food Sci..

[23] Cuthbertson D., Andrews P.K., Reganold J.P., Davies N.M., Lange B.M. (2012). Utility of metabolomics toward assessing the metabolic basis of quality traits in apple fruit with an emphasis on antioxidants. J. Agric. Food Chem..

[24] Du Y., Li X., Xiong X., Cai X., Ren X., Kong Q. (2021). An investigation on polyphenol composition and content in skin of grape (*Vitis vinifera* L. cv. Hutai No. 8) fruit during ripening by UHPLC-MS2 technology combined with multivariate statistical analysis. Food Biosci..

[25] Keller M. (2020). The Science of Grapevines.

[26] Liang Z., Sang M., Fan P., Wu B., Wang L., Yang S., Li S. (2011). CIELAB coordinates in response to berry skin anthocyanins and their composition in Vitis. J. Food Sci..

[27] Geroyiannaki M., Komaitis M., Stavrakas D., Polysiou M., Athanasopoulos P., Spanos M. (2007). Evaluation of acetaldehyde and methanol in greek traditional alcoholic beverages from varietal fermented grape pomaces (*Vitis vinifera* L.). Food Control.

[28] Biniari K., Xenaki M., Daskalakis I., Rusjan D., Bouza D., Stavrakaki M. (2020). Polyphenolic compounds and antioxidants of skin and berry grapes of Greek Vitis vinifera cultivars in relation to climate conditions. Food Chem..

[29] Boss P., Davies C., Roubelakis-Angelakis K.A. (2001). Molecular biology of sugar and anthocyanin accumulation in grape berries. Molecular Biology & Biotechnology of the Grapevine.

[30] Hasan M., Bae H. (2017). An Overview of Stress-Induced Resveratrol Synthesis in Grapes: Perspectives for Resveratrol-Enriched Grape Products. Molecules.

[31] Melzoch K., Hanzlíková I., Filip V., Buckiová D., Šmidrkal J. (2001). Resveratrol in parts of vine and wine originating from Bohemian and Moravian vineyard regions. Agric. Conspec. Sci..

[32] Frémont L. (2000). Biological effects of resveratrol. Life Sci..

[33] Samah M., Sahar S., Khaled A., Hoda M. (2012). Phenolic compounds and antioxidant activity of white, red, black grape skin and white grape seeds. Life Sci. J..

[34] Brighenti E., Casagrande K., Cardoso P.Z., Pasa M.d.S., Ciotta M.N., Brighenti A.F. (2017). Total polyphenols contents in different grapevine varieties in highlands of southern brazil. BIO Web Conf..

[35] Lingua M.S., Fabani M.P., Wunderlin D.A., Baroni M.V. (2016). From grape to wine: Changes in phenolic composition and its influence on antioxidant activity. Food Chem..

[36] DeBolt S., Cook D.R., Ford C.M. (2006). L-Tartaric acid synthesis from vitamin C in higher plants. Proc. Natl. Acad. Sci. USA.

[37] Razungles A., Bayonove C.L., Cordonnier R.E., Sapis J.C. (1988). Grape Carotenoids: Changes During the Maturation Period and Localization in Mature Berries. Am. J. Enol. Vitic..

[38] Doshi P., Adsule P., Banerjee K. (2006). Phenolic composition and antioxidant activity in grapevine parts and berries (*Vitis vinifera* L.) cv. Kishmish Chornyi (Sharad Seedless) during maturation. Int. J. Food Sci. Technol..

[39] Benbouguerra N., Richard T., Saucier C., Garcia F. (2020). Voltammetric Behavior, Flavanol and Anthocyanin Contents, and Antioxidant Capacity of Grape Skins and Seeds during Ripening (Vitis vinifera var. Merlot, Tannat, and Syrah). Antioxidants.

[40] Kallithraka S., Aliaj L., Makris D.P., Kefalas P. (2009). Anthocyanin profiles of major red grape (Vitis vinifera L.) varieties cultivated in Greece and their relationship with in vitro antioxidant characteristics. Int. J. Food Sci. Technol..

[41] Conde C., Silva P., Fontes N., Dias A.C.P., Tavares R.M., Sousa M.J., Agasse A., Delrot S., Gerós H. (2007). Biochemical changes throughout grape berry development and fruit and wine quality. Food.

[42] Lanaridis P., Bena-Tzourou I. (1997). Study of anthocyanins’ variations during the ripening of five vine red varieties cultivated in Greece. OENO One.

[43] Kyraleou M., Gkanidi E., Koundouras S., Kallithraka S. (2019). Tannin content and antioxidant capacity of five Greek red grape varieties. Vitis-J. Grapevine Res.

[44] Vitrac X., Larronde F., Krisa S., Decendit A., Deffieux G., Mérillon J.-M. (2000). Sugar sensing and Ca2+–calmodulin requirement in Vitis vinifera cells producing anthocyanins. Phytochemistry.

[45] Kennedy J.A., Saucier C., Glories Y. (2006). Grape and wine phenolics: History and perspective. Am. J. Enol. Vitic..

[46] Adams D.O. (2006). Phenolics and ripening in grape berries. Am. J. Enol. Vitic..

[47] Kennedy J.A., Matthews M.A., Waterhouse A.L. (2002). Effect of Maturity and Vine Water Status on Grape Skin and Wine Flavonoids. Am. J. Enol. Vitic.

[48] Downey M., Harvey J., Robinson S. (2003). Synthesis of flavonols and expression of flavonol synthase genes in the developing grape berries of Shiraz and Chardonnay (*Vitis vinifera* L.). Aust. J. Grape Wine Res..

[49] Vincenzi S., Tomasi D., Gaiotti F., Lovat L., Giacosa S., Torchio F., Segade S.R., Rolle L. (2013). Comparative study of the resveratrol content of twenty-one Italian red grape varieties. S. Afr. J. Enol. Vitic..

[50] (2007). International Commission on Illumination. Standard Illuminants for Colorimetry.

[51] Singleton V.L., Orthofer R., Lamuela-Raventós R.M. (1999). Analysis of total phenols and other oxidation substrates and antioxidants by means of folin-ciocalteu reagent. Methods Enzymol.

[52] Poudel P.R., Tamura H., Kataoka I., Mochioka R. (2008). Phenolic compounds and antioxidant activities of skins and seeds of five wild grapes and two hybrids native to Japan. J. Food Compost. Anal..

[53] Arnous A., Makris D.P., Kefalas P. (2001). Effect of Principal Polyphenolic Components in Relation to Antioxidant Characteristics of Aged Red Wines. J. Agric. Food Chem..

[54] Cvetković Ž.S., Nikolić V.D., Savić I.M., Savić-Gajić I.M., Nikolić L.B. (2015). Development and validation of an RP-HPLC method for quantification of trans-resveratrol in the plant extracts. Hem. Ind..

